# Spatial and temporal patterns of genetic diversity in *Bombus terrestris* populations of the Iberian Peninsula and their conservation implications

**DOI:** 10.1038/s41598-021-01778-2

**Published:** 2021-11-18

**Authors:** Diego Cejas, Pilar De la Rúa, Concepción Ornosa, Denis Michez, Irene Muñoz

**Affiliations:** 1 Área de Biología Animal, Departamento de Zoología y Antropología Física, Facultad de Veterinaria, Universidad de Murcia, 30100 Murcia, Spain; 2 Laboratory of Zoology, Research Institute for Biosciences, University of Mons, Place du parc 20, 7000 Mons, Belgium; 3 Departamento de Biodiversidad, Ecología y Evolución, Facultad de Ciencias Biológicas, Universidad Complutense, 28040 Madrid, Spain

**Keywords:** Conservation biology, Population dynamics, Population genetics, Genetic hybridization, Population genetics

## Abstract

The bumblebee *Bombus terrestris* is used worldwide for crop pollination. Despite its positive impact on crop yield, it has become a widespread threat to biodiversity due to its interactions with local bumblebee populations. Commercial subspecies introduced to the Iberian Peninsula since the 1990s without any regulation have colonized the environment, with evidence of naturalization and introgression with the endemic subspecies *Bombus terrestris lusitanicus*. We have used mitochondrial and nuclear genetic data to describe the current genetic diversity of the Iberian population and to estimate the expansion of commercial bumblebees. Samples from the natural distribution range of the commercial subspecies, the natural intergradation area between the two subspecies and from a period prior to the use of commercial colonies (i.e., before the 1990s) have been used for comparison. Our results show that the mitochondrial haplotype of the commercial breeds has spread throughout the territory, which, together with subtle changes observed in the nuclear genetic diversity of the populations, indicates that hybridization and consequent introgression are occurring in most of the peninsula. It is, therefore, necessary to improve the existing legislation concerning the management and exportation of commercial bumblebees to conserve locally adapted populations.

## Introduction

In the context of the current human-induced biodiversity crisis^1^, scientific knowledge about the drivers of decline and how they affect organisms is necessary to propose adequate conservation measures^2^. In the last decades, given their economic and environmental importance, concern for the welfare of pollinators has risen^3^. Many studies have explored the main potential causes of their decline^4^. In addition to those identified as major causes (e.g., pesticides and loss of habitat^5^), anthropic translocations of domesticated taxa like honey bees or bumblebees within their natural ranges are receiving more attention^6,7^. Such human-mediated movement may harm other pollinators through competition for resources and the spread of parasites (e.g., microsporidia and trypanosomatids^8–10^). More research on these effects is needed to encourage regulatory authorities to implement scientifically supported conservation measures.

The West Palearctic bumblebee species *Bombus terrestris* (Linnaeus, 1758) began to be used commercially in greenhouses for crop pollination in the 1980s in Belgium, and the subspecies *B. t. terrestris* and *B. t. dalmatinus* Dalla Torre, 1882 are now widely used in artificial pollination^11^. Commercial breeds can escape from greenhouses into natural habitats when they seek floral resources^12^ or nesting places and can colonize suitable environments due to their high thermic resistance, generalist diet and wide climatic tolerance^13,14^. The exportation of *Bombus terrestris* outside its Palearctic distribution range has made this bumblebee an invasive species^15,16^, driving native species to local decline or extinction due to competition, pathogen spillover and reproductive interference^2,9,17^.

Many countries have already taken legislative measures to manage this situation. In Japan, *B. terrestris* was classified as an invasive species in 2006 and its importation is now severely restricted^17^. In Argentina, the importation of *B. terrestris* and *B. impatiens* Cresson, 1863 is also strongly regulated to protect the native South American species *B. dahlbomii* Guérin-Méneville, 1835^16^, although commercial species can still spread from Chile^15^. Within the natural distribution range of the species, specific regulations to protect endemic subspecies of *B. terrestris* have also been approved, as commercial breeds could become a potential competition to the wild populations based on their characteristics (e.g., higher gyne production, greater foraging ability and larger colony size)^8^. Norway and the Canary Islands have strict importation policies on foreign bumblebees^11^, while in the United Kingdom the trade of non-native subspecies is restricted, favouring the use of the endemic subspecies *B. t. audax* (Harris, 1776)^9^. However, no regulation has been enacted to protect the endemic subspecies *B. t. lusitanicus* (Krüger, 1956) in the Iberian Peninsula, where there is a high density of greenhouse agriculture that use bumblebees as managed pollinators (Supplementary Fig. S1, online).

*B. terrestris* presents eight subspecies based on colour patterns, distribution and pheromonal analysis^18,19^*. B. t. lusitanicus, B. t. terrestris* and *B. t. dalmatinus* are described with a yellow and black band pattern and a white tail at the end of the abdomen. However, *B. t. lusitanicus* also presents a variable colouration of ferruginous brown in the ventral area of the gaster, pleurae and corbiculae that allows its differentiation^18,20^.

The natural distribution range of *B. t. lusitanicus* includes the Iberian Peninsula, a current hotspot of biodiversity after its role as a glacial refugium in the Quaternary period^21^, and the Pyrenees and south of France, where it overlaps with the central European subspecies *B. t. terrestris*. The similar composition of the cephalic labial gland secretions of the two subspecies^13^ facilitates gyne preference towards interbreeding and, therefore, a natural intergradation area occurs in the distribution overlap^18,20^. The colouring of hybrid specimens varies, but they typically show a variable mixture of light and dark pubescence on the ventral area of the gaster, pleurae and, especially, the legs and corbiculae.

Since the establishment of rearing companies in the 1990s in the region of Andalucia in southern Spain, there are records of the naturalization of managed commercial bumblebees in the environment^22^. Nowadays, a new anthropic intergradation area has developed in the south of the peninsula, where hybridization events and introgression with wild populations have been confirmed with genetic evidence^23–25^.

To preserve endemic taxa, the genetic diversity and structure of local populations must first be investigated^26^. Conservation genetics provides a theoretical framework of the appropriate methodologies to reveal differences between taxa^27^, populations^28^ and historical data^29^. The use of mitochondrial and nuclear markers in population genetics is advisable due to the different inheritance mechanisms of these genomes^30^, especially in haplodiploid species like bees that can show a strong mitochondrial bias in introgression rates^31^.

Mitochondrial DNA presents certain implications because of its maternal inheritance. In bumblebees, mitochondrial DNA analysis has confirmed the presence of exotic queens in the environment^23^ and of drifter workers that can lay unfertilized eggs in foreign colonies, resulting in male adults^10^. Moreover, based on empirical data, the maternal genotype is considered decisive for the inheritance of the colony traits^32^. Finally, the mitochondrial haplotype of the studied subspecies have already been characterized^23^. Alternatively, nuclear markers such as microsatellites are a validated population genetics tool to study the genetic diversity and structure of populations in the genus^7,29,33^.

Both mitochondrial and nuclear markers were analysed to estimate genetic diversity parameters and clustering patterns across *Bombus terrestris* populations in the Iberian Peninsula. To study the effect of hybridization, some analyses were repeated after filtering hybrid individuals (based on morphological determination and molecular analysis) to identify differential changes in the genetic diversity of populations. Male diploidy rates (2n**♂)** were also determined to estimate the inbreeding in bumblebee populations^34,35^. In haplodiploid species such as *B. terrestris*, male diploidy is caused by homozygosis at the sex determination locus (sl-CSD) in diploid individuals, which would develop into females if they were heterozygous^36^. The presence of diploid males implies a reduction in the number of workers, which can negatively affect the fitness of the colony^28^. Although male diploidy occurs naturally^37^, high rates have been linked with declines in effective population size and low genetic diversity^38^ and therefore would be expected to appear at a lower rate in hybrid populations.

This research aims to study the genetic diversity and structure of the Iberian *B. terrestris* populations from a broader perspective on both spatial (i.e., Iberian Peninsula) and temporal (before and after the 1990s) scales, taking into account the dispersion of commercial breeds (i.e., central European subspecies) into the wild and potential introgression events between wild and commercial populations. We hypothesize that, due to the dispersion of commercial subspecies from greenhouses, hybridization with the wild populations of the endemic subspecies is not limited to the south and east of the Iberian Peninsula^23–25,39^ and introgression is occurring across the territory. These events would have a more pronounced impact on the genetic diversity of the populations in the south of the peninsula, where many bumblebee breeding companies provide pollination services to the high quantity of greenhouse crops that depend on bumblebees^40^.

## Results

### Subspecies and hybrid distribution based on morphology

Of the 594 studied individuals, 39 were discarded as eight corresponded to a different species after BLAST analyses (seven *B. lucorum* [Linnaeus, 1761] and one *B. soroeensis* [Fabricius, 1777]), and 31 from pinned collections had low-quality DNA extractions. Of the remaining 555 individuals (365 females and 190 males), 487 were morphologically identified as *B. t. lusitanicus* (87.75%)*,* 58 as *B. t. terrestris* (10.45%) and 10 as morphological hybrids (1.8%). Within the Iberian sampling, *B. t. terrestris* individuals and most hybrids were found in the south of the peninsula and in the Pyrenees (Table 1), (i.e. in the two main detected areas of intergradation). However, two hybrids were found in Sierra de Guadarrama (SP_SG3, central peninsula), where two subspecies are not believed to be in contact.

**Table 1:** Subspecies identification of *B. terrestris* individuals and hybrids detected per location (see Supplementary Table S1 online for location codes).

Location	Subspecies	N	♀	hybr	♂ (2n)	hybr	% hybr
SP_VI	*lusitanicus*	3			3 (1)		0
SP_PO	*lusitanicus*	11	7		4		0
SP_BU	*lusitanicus*	7	7	2			28.57
SP_PA	*lusitanicus*	5	5				0
SP_SO	*lusitanicus*	11	9	2	2		18.18
PT_BR	*lusitanicus*	7	3		4		0
PT_VC	*lusitanicus*	14	14	1			7.14
SP_SG1	*lusitanicus*	10	10	1			10.00
SP_SG2	*lusitanicus*	138	67	6	71 (5)	9	10.87
SP_SG3	*lusitanicus*	33	23	2	10	2	17.14
	hybrids*	2	1		1		
SP_MA	*lusitanicus*	12	9		3 (2)		0
SP_MU1	*lusitanicus*	23	16	4	7 (2)	3	30.43
SP_MU2	*lusitanicus*	25	18	7	7 (2)	4	44.00
SP_HU	*lusitanicus*	7	2	1	5	2	42.86
SP_SN1	*lusitanicus*	16	8	1	8	1	17.65
	*terrestris*	1			1	1	
SP_SN2	*lusitanicus*	40	15	3	25	4	29.17
	hybrids*	4			4		
	*terrestris*	4	4	3			
SP_SN3	*lusitanicus*	51	27	6	24 (1)	10	35.94
	*terrestris*	13	11	5	2	2	
SP + PT	lusitanicus	413	240	36	173 (13)	35	20.13
	hybrids*	6	1		5		
	terrestris	18	15	8	3	3	
REF_BL	terrestris	12	12				0
REF_FR	terrestris	23	18	3	5		13.04
PN_AM	lusitanicus	14	14	5			40.00
	terrestris	1	1	1			
PN_EY	lusitanicus	15	15	6			47.37
	terrestris	4	4	3			
PN_JA	lusitanicus	1	1	1			100.00
	hybrids*	4	4	4			
PN	lusitanicus	30	30	12			51.28
	hybrids*	4	4	4			
	terrestris	5	5	4			
REF_SP80	lusitanicus	44	40	1	4	0	2.27

Hybrids have been identified either morphologically (labelled with * in the subspecies column) or through discrepancies between morphologic identification and mitochondrial haplotype (hybr. column). Samples have been separated by sex, showing the number of diploid males (2n) in parentheses. The percentage of hybrid individuals (morphological and mitochondrial) is shown by sampling site. N = sample size.

### Subspecies and hybrid distribution based on mitochondrial haplotypes

Mitochondrial haplotype information was obtained for the 555 morphologically described individuals. The mitochondrial haplotype was determined by the *nad2*-RFLP approach in 433 individuals (78.01%), by sequencing the *Bter_rrnL* fragment in 33 individuals (5.95%) and by the *16S* fragment in 89 individuals (16.04%) from previous studies^23,39^.

**Figure 1:**
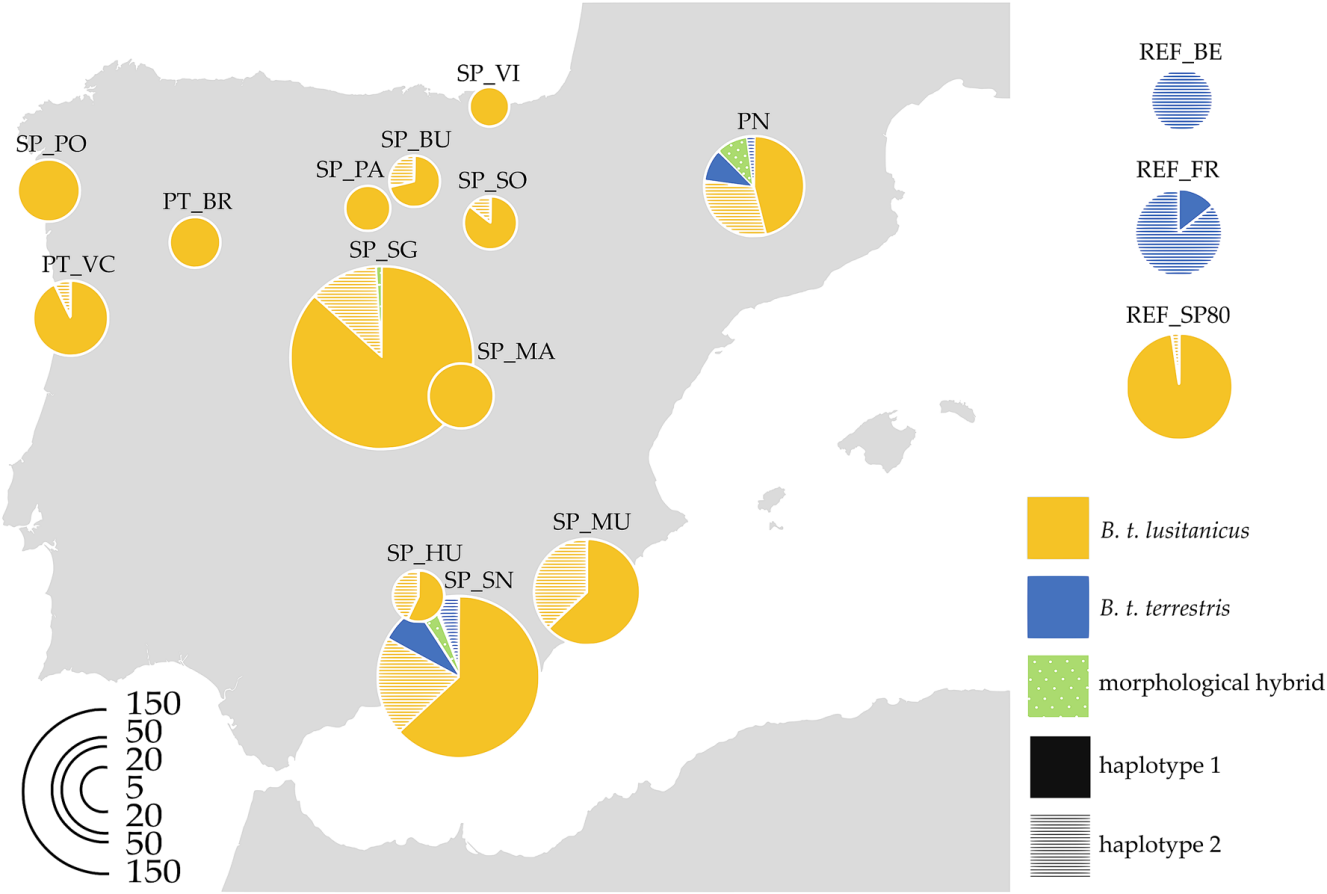
*Bombus terrestris* subspecies and haplotype distribution in the Iberian Peninsula based on their morphologic traits and the genetic haplotypes within the mitochondrial genes *nad2* and *rrnL*. Subspecies information is shown by colour: yellow for *B. t. lusitanicus* and blue for *B. t. terrestris*; haplotype information is shown by pattern: plain for haplotype 1 (Iberian) and striped for haplotype 2 (central European). Morphological hybrids are represented by green and a spotted pattern. The data of the reference populations are shown on the top right. The radius of the circles is proportional to the number of individuals analysed in each location. The data from nearby locations have been merged for readability. This map has been adapted with Inkscape 0.92 from https://commons.wikimedia.org/wiki/File:Iberian_Peninsula_location_map.svg, licensed under the Attribution-ShareAlike 3.0 Unported license (https://creativecommons.org/licenses/by-sa/3.0/).

Two haplotypes were detected: a haplotype more frequent in the Spanish and Portuguese populations (the Iberian haplotype, SP + PT: 81.70%) and one more frequent in the Belgian and French populations (the central-European haplotype, REF_BL: 100%; REF_FR: 86.96%) (Fig. 1). A different haplotype from that expected based on individuals’ morphology (discrepant hybrid) was detected in 20.13% of the analysed individuals in the peninsula. *B. t. lusitanicus* individuals with the central-European haplotype (discrepant hybrids) were found in 12 of the 17 Iberian locations sampled (0% to 44%) and the Pyrenees (18.74%). 61.1% of the naturalized *B. t. terrestris* found in the south of the peninsula bore the Iberian haplotype. Higher percentages of discrepant hybrids were found in the two intergradation areas: the Pyrenees (PN_JA: 100%; PN_EY: 47.37%; PN_AM: 40.00%) and the southern peninsula (SP_MU2: 44.00%; SP_SN3: 35.94%), while three individuals from France presented the Iberian haplotype (REF_FR: 13.04%) and no hybrids were found in the Belgian reference group. In the historical population, the central-European haplotype was found only in one individual close to the intergradation area of the Pyrenees (Iberian haplotype in REF_SP80: 97.73%).

### Microsatellite data validation

Of the ten microsatellite loci amplified, locus B10 was excluded due to a low amplification rate and difficulty in scoring. In addition, 27 individuals with > 30% missing data were removed from further analyses and 18 females were also removed after sibship inference analysis, leaving a total of 320 female individuals for population analysis. Null alleles due to homozygote excess were only found in the Iberian populations (SP + PT) and the historical reference (REF_SP80) at low frequencies (Oosterhout > 0.25%). Most loci presented a statistically significant deviation from HWE expectations due to heterozygote deficit. None of the microsatellite loci analysed showed significant linkage disequilibrium.

### Subspecies and hybrid distribution based on microsatellite markers

Microsatellite information was only considered from locations with more than ten genotyped individuals (N = 254; PT_VC, SP_SG2, SP_SG3, SP_MU1, SP_MU2, SP_SN3, PN_EY, REF_SP80, REF_BL and REF_FR).

**Figure 2:**
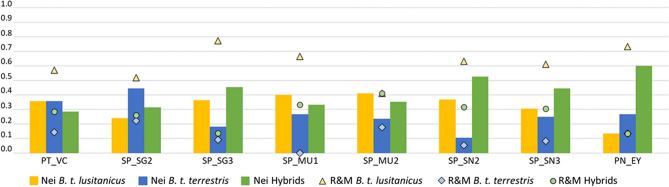
Distribution of highest probability subspecies or hybrid assignments of Iberian bumblebee individuals originating from each geographical location using the resampling procedure in GeneClass 2.0 by Nei’s standard distance method (Nei, bars), and by Rannala & Mountain’s Bayesian method (R&M, points).

A total of 19 individuals from the historical population (REF_SP80) were classified as reference *B. t. lusitanicus* and 17 from Belgium and France (REF_BL and REF_FR) as reference *B. t. terrestris* by the assignment test using GenAlEx*.* The presence of hybridization was corroborated with microsatellite analysis. The GeneClass assignment test using Nei’s standard distance^41^ and the Bayesian method^42^ allowed us to observe introgression in all the Iberian localities analysed (Fig. 2). Comparing both methods, the Bayesian method assigned a greated percentage of individuals to the *lusitanicus* subspecies. However, despite the discrepancies in assignment between methods, likely reflecting their statistical differences and the difficulty of reliably detecting hybrids^43^, both methods approximately coincide in the percentages of hybrids obtained at PT_VC, SP_SG2, SP_MU1 and SP_MU2.

Assignment analysis showed that the percentage of hybrids identified at the nuclear level is higher than that detected with morphological determination and mitochondrial haplotype methods, especially in areas with percentages of hybrids higher than 20.00%. A positive correlation was observed (r^2^ = 0.593; *p* = 0.033) between the percentage of hybrids found by mitochondrial haplotype and by nuclear genetic parameters (Supplementary Fig. S2, online).

### Genetic diversity and effect of hybridization

Allelic richness (Ar) ranged from 4.27 to 4.63 across the Iberian populations (Table 2). The lowest allelic richness was found in the Pyrenees (PN_EY: 4.16) and the highest value was found in Normandy (Ref_FR: 4.70). More private alleles were found in the overall Iberian population. Genetic diversity estimated from expected heterozygosity (*H*_E_) values varied from 0.630 to 0.716 (observed in the Pyrenean PN_EY and French Ref_FR populations, respectively). The fixation index (Fis) showed signs of inbreeding in both the Iberian and the historical reference populations.

**Table 2:** Allelic richness (Ar), private allelic richness (pAr), observed heterozygosity (Ho), expected heterozygosity (*H*_E_) and inbreeding index (Fis) were calculated independently for each population based on the genotypes of female individuals.

Location	N		Ar		pAr		Ho		*H*_E_		Fis	
	*hIN*	*hOUT*	*hIN*	*hOUT*	*hIN*	*hOUT*	*hIN*	*hOUT*	*hIN*	*hOUT*	*hIN*	*hOUT*
PT_VC	14	13	4.45	4.43	0.19	0.16	0.586	0.572	0.659	0.660	0.148	0.174
SP_SG2	54	49	4.29	4.32	0.11	0.10	0.532	0.524	0.671	0.672	0.217	0.229
SP_SG3	22	19	4.27	4.21	0.13	0.10	0.590	0.576	0.680	0.665	0.156	0.162
SP_MU1	15	11	4.63	4.57	0.30	0.31	0.708	0.733	0.700	0.687	0.033	− 0.01
SP_MU2	17	11	4.44	4.60	0.12	0.09	0.570	0.619	0.698	0.690	0.215	0.157
SP_SN2	19	12	4.45	4.42	0.11	0.13	0.570	0.624	0.687	0.662	0.197	0.101
SP_SN3	36	20	4.50	4.37	0.16	0.16	0.629	0.611	0.701	0.683	0.117	0.131
SP + PT	177	135	4.46	4.43	0.39	0.40	0.587	0.584	0.701	0.696	0.166	0.165
REF_BL	11	11	4.48	4.48	0.13	0.18	0.717	0.717	0.661	0.661	− 0.036	− 0.036
REF_FR	17	14	4.70	4.62	0.23	0.15	0.739	0.778	0.716	0.707	− 0.001	− 0.064
PN_EY	15	8	4.16	3.85	0.11	0.13	0.588	0.644	0.630	0.589	0.107	0.07
REF_SP80	34	33	4.69	4.66	0.36	0.37	0.498	0.488	0.710	0.706	0.314	0.326

N = population size. hIN includes all individuals while hOUT excludes hybrid individuals (morphological and mitochondrial).

To study introgression at the nuclear level in the Iberian populations, population parameters (dataset with hybrids, hIN) were compared with those obtained after the removal of individuals labelled as hybrids (whether by morphological description or because of their mitochondrial haplotype) in each location (dataset without hybrids, hOUT) (Table 2). The Wilcoxon signed-rank test showed a strong variation in the expected heterozygosity values after the removal of the hybrids, although it was not significant after Bonferroni correction (*p* = 0.014, V = 52). The results after random removal (hIN/rOUT) (*p* = 0.082, V = 45) suggest that the decrease of *H*_E_ in hOUT might not be due to the mere removal of individuals (Table 3), but to the hybrid identity of the removed individuals.

**Table 3:** Wilcoxon signed-rank test for related samples.

	N	Ar	pAr	Ho	He	Fis
***hIN/hOUT***						
V Wilcoxon	55	43	32	15	52	40
*p*-value	0.006*	0.125	0.682	0.221	0.014	0.221
***hIN/rOUT***						
V Wilcoxon	55	32	32	17	45	30.5
*p*-value	0.006*	0.684	0.679	0.944	0.082	0.374

hIN/hOUT: Population parameters from the whole dataset (hIN) were compared with those obtained without hybrids (hOUT). To avoid type I errors, the Wilcoxon test was repeated, comparing the results with those obtained after the random removal of individuals (hIN/rOUT). N = population size, Ar = allelic richness, pAr = private allelic richness, Ho = observed heterozygosity, He = expected heterozygosity, Fis = inbreeding index, * = *p* < 0.008.

**Figure 3:**
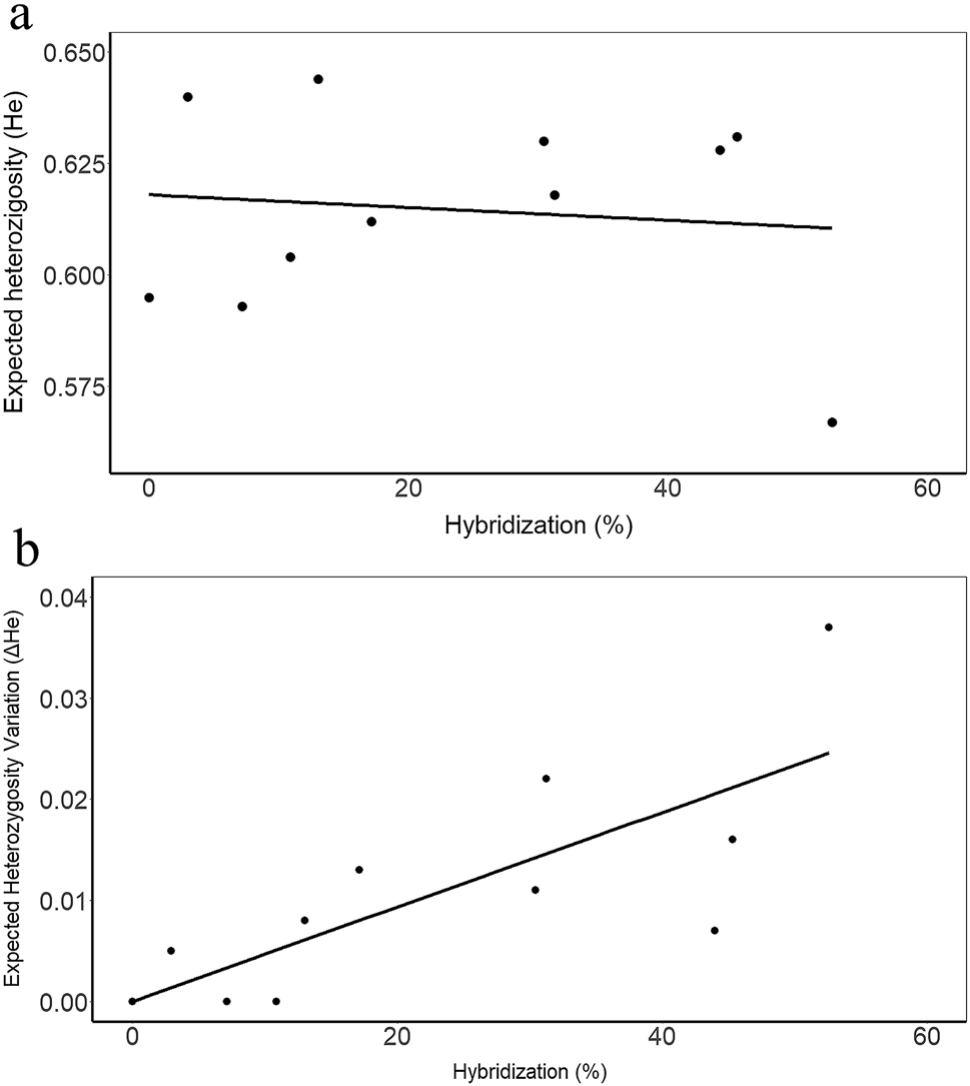
Linear regressions between (**a**) genetic diversity (*H*_E_) or (**b**) variation in genetic diversity (Δ*H*_E_) and the percentage of hybrids observed in each population. Genetic diversity does not decrease significantly as the percentage of hybrids rises (r^2^ = 0.012, *p* = 0.741), but when the percentage of hybrids increases, the variation in genetic diversity increases as well (r^2^ = 0.593, *p* = 0.005).

Linear regressions showed that genetic diversity values did not depend directly on the percentage of hybrid individuals in each location (r^2^ = 0.012; *p* = 0.741) and were likely affected by other variables. However, the variation in *H*_E_ due to the presence of hybrid individuals (Δ*H*_E_ = *H*_E_*hIN* − *H*_E_*hOUT*) does depend significantly on the percentage of hybrids present in each location (r^2^ = 0.593; *p* = 0.005) (Fig. 3).

### Genetic structure

**Figure 4:**
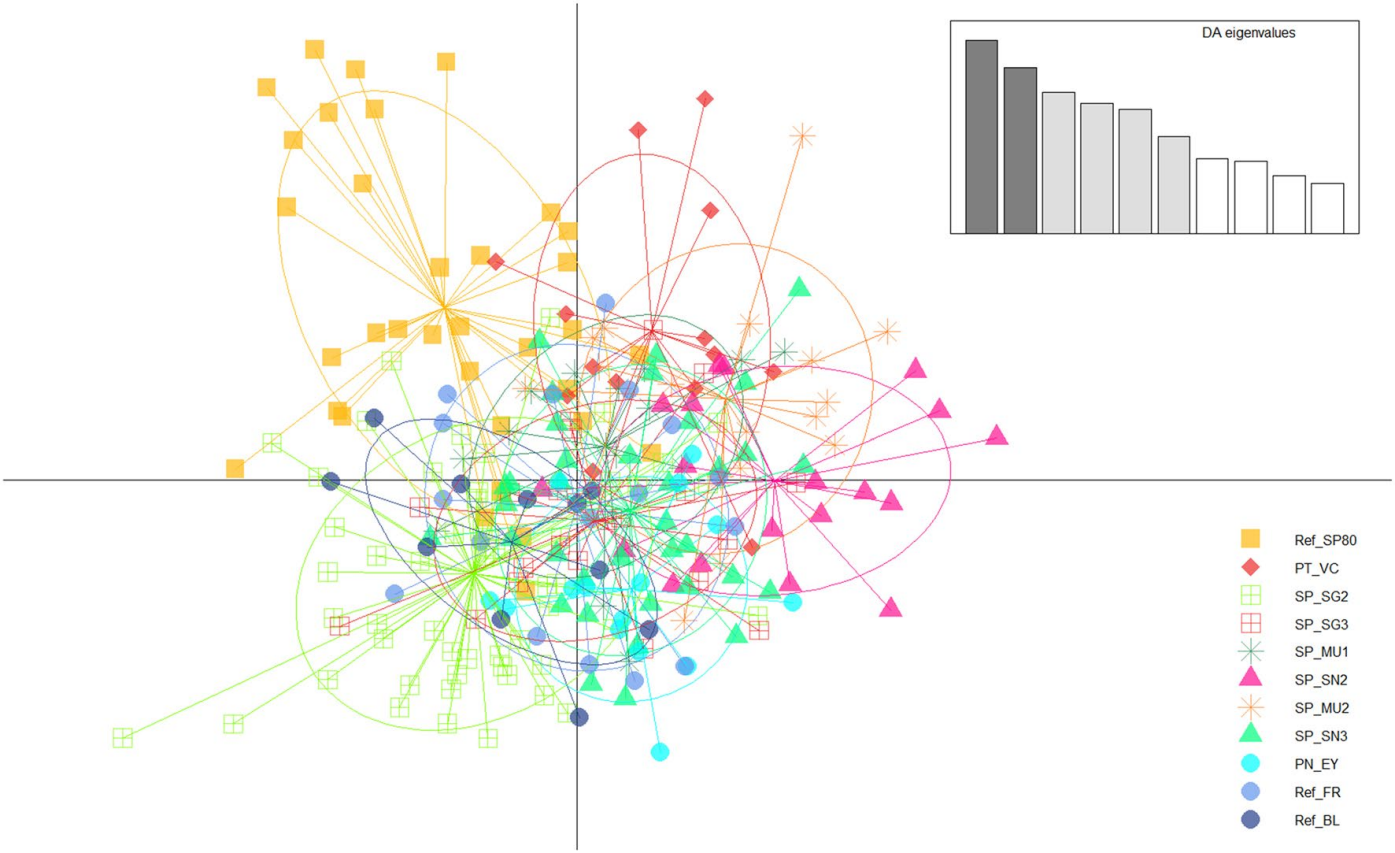
Scatter plot from discriminant analysis of principal components. 80 principal components (PC) were selected after α-score estimations to avoid overfitting. Chosen discriminant analysis eigenvalues are depicted in the top right of the plot. Ellipses indicate the 95% confidence interval of assignment, yellow squares identify bumblebee individuals collected before the 1990s, orange rhombuses represent Portuguese samples, green represents individuals from Sierra de Guadarrama, pink represents individuals from the Sierra Nevada and blue represents samples from outside the Iberian Peninsula, as detailed in the figure legend at the bottom right.

Structure Harvester showed K = 4 as the most likely number of clusters (Supplementary Fig. S3, online), although this result is probably a consequence of the limitations of the program, which cannot detect values of K = 1^44^. However, K = 2 was also studied to investigate possible structuration due to the subspecies (*lusitanicus* and *terrestris*) present (Supplementary Fig. S4, online). Visualization of Structure results indicates approximately equal contributions to each population (K), implying that the true value of K is indeed 1, with no clear structuring due to subspecies. The *find.clusters* function from the package adegenet 2.1.1 showed that BIC was minimised at K = 4, with K = 2 presenting the largest increase in BIC (Supplementary Fig. S5, online). Clusters observed at K = 2 to K = 4 were not related either to the subspecies identification or population (Supplementary Fig. S6, online). A final analysis in which populations were selected as clusters (Fig. 4) showed an overlapping distribution in the axis, indicating a low degree of genetic differentiation and corroborating the Structure results. However, the Iberian population from the historical reference group REF_SP80 presented a higher degree of clustering than the other populations.

### Diploid male detection

A total of 13 diploid males were found out of the 181 male individuals sampled in the Iberian Peninsula (Table 1): one in the north (SP_VI: 1, ϕ = 0.3), seven in the central area (SP_SG2: 5, ϕ = 0.07; SP_MA: 2, ϕ = 0.66) and five in the south (SP_MU1: 2, ϕ = 0.29; SP_MU2: 2, ϕ = 0.29; SP_SN3: 1, ϕ = 0.04), with an overall ϕ for the Iberian Peninsula of 0.07. A male from the locality SP_SG2 was identified as a triploid (3n) after amplifying each microsatellite locus individually.

## Discussion

This research aimed to study the genetic diversity of the Iberian *B*. *terrestris* populations under threat of introgression from managed non-native populations at both the spatial and temporal scales. Our results, based on extensive sampling throughout the Iberian Peninsula (Spain and Portugal), indicate that the mitochondrial haplotype associated with central European populations of *B. terrestris* has expanded beyond both the natural intergradation area (i.e., the Pyrenees) and the anthropic one (i.e., the southeast region), reaching most of the north–south gradient with some exceptions in the northwest. These results contrast with the haplotype distribution in historical populations (i.e., REF_SP80, specimens collected in Spain 40 years ago) in which all individuals presented the Iberian haplotype, except one individual detected close to the natural intergradation area in the Pyrenees, where natural hybridization occurs. These results support the idea that the endemic Iberian haplotype was predominant in the territory and that, due to hybridization and introgression events with naturalized commercial breeds, the central European haplotype has recently spread into the environment. This change in the haplotype distribution of the peninsula is a first signal of how this expansion is modifying the genetic pool of the populations, which may soon develop into losses of local adaptation^6,45^. Another hypothesis to explain the present genetic structure could be migration, although unlikely. In the context of climate change, the only plausible hypothesis is a migration from the Southern area to the north^1^. However, based on our knowledge, the European subspecies was never recorded in the Southern region of Spain and we did not detect any individuals with the central European haplotype in the historical data set but one, close to the natural intergradation area in Pyrenees.

Our results expand the current knowledge about the genetic integrity of *B. t. lusitanicus* in the Iberian Peninsula. Previous records of *B. t. terrestris* and hybrid individuals have occurred only in the south^23,25,39^ and west^24^ (putative genetic hybrids) of the Iberian Peninsula, whereas we have found evidence of introgression in most of the territory (see Table 1 and Fig. 1). The presence of the central European haplotype in the central area of the peninsula, where the subspecies *B. t. terrestris* has not been detected, could be due to the dispersion of individuals from areas where commercial breeds are being used (greenhouses in the region or an expansion from the south), as *B. terrestris* dispersion can be assisted by strong winds to cross long distances^15,33^. On the other hand, in the most northwestern localities sampled (SP_PO, SP_BR and SP_PA), the central European haplotype was not found, which suggests that these populations of *B. t. lusitanicus* are less affected by introgression, probably due to a lower density of greenhouses in the area, as well as a larger distance from the two main areas of intergradation^40^ (Supplementary Fig. S1 Online).

This study confirms that the inclusion of samples from old collections is crucial to assess the evolution of the genetic diversity of local populations, as has been done before in other species of the genus *Bombus* (Latreille, 1802)^29^. Furthermore, the use of old samples as historical references in population genetics will be especially important in subsequent years, as we monitor the expected changes in the distribution of not only *B. terrestris* subspecies but also other organisms that are affected by climatic change and anthropisation. The distribution range of *B. t. lusitanicus*, instead of shifting or reducing, is expanding, both in altitude and latitude^13,18,46^, while the populations of *B. t. audax* in the UK have become bivoltine^7^. Given the subspecies’ similar resistance to heat stress^14^, further changes due to climatic change are expected to occur. Even under these conditions, although we must assume that hybridization may play a key role in evolution^47,48^, there is a consensus that human-induced introgression of non‐indigenous organisms harms native gene pools^45,49,50^.

As previous studies on the genetic diversity of the species have reported, the heterozygosity values obtained in this analysis, the assignment test (GeneClass) and the clusters inferred (Structure and DAPC) suggest an intense gene flow among *B. terrestris* populations, which leads to a reduced population structure in a continuum that is not only peninsular but also continental^33,51,52^. When comparing the Iberian populations with their historical reference, only slight variations can be observed in the allelic frequencies, although the data show an overall decrease in allelic richness and expected heterozygosity values. Conversely, the Wilcoxon test suggests that hybridization and introgression events are affecting the native populations by increasing the genetic diversity of those populations with hybrid individuals, which is expected from the admixture of different gene pools^45^. Given the low structure of bumblebee populations, the effect of this change is difficult to measure, but if this dynamic continues, the loss of endemicity and increased homogenization of European populations will be recurring concerns in the future.

The analysis of genetic diversity in the Iberian Peninsula showed the lowest values in the central peninsular populations (Sierra de Guadarrama: SP_SG2, SP_SG3), where the highest number of diploid males and one triploid male were found. These results could represent a sign of a potential threat of inbreeding depression in the populations^35^, although it has been previously discussed that *B. terrestris* is able to withstand inbreeding in its populations^56^. In this sense, *B. terrestris* does not show mating preferences between related and unrelated individuals^35^, so there are no mechanisms preventing diploidy or even triploidy in males^53^. However, these results should be taken into account in future studies on the species, as the ploidy values obtained in this study were higher than those of populations of endangered species such as *B. florilegus* Panfilov, 1956 (2.7%)^54^ or *B. muscorum* Linnaeus, 1758 (5%)^55^.

Given that *B. terrestris* can escape from greenhouses^23,24^ and the great expansion capacity of the managed populations in the environment^15^, we emphasize the importance of the propagation of the commercial *B. terrestris* subspecies from greenhouses across the Iberian Peninsula as a driver of population change. Moreover, the inadequate management of colonies by breeding companies and farmers after colony use due to a lack of information about the consequences of their dispersal in the environment is another little-addressed factor contributing to the emergence and dispersion of commercial breeds (implied by the presence of the central European haplotype) in the environment. Some of the mispractices that occur in the territory include the outdoor use of colonies, incomplete removal or abandonment of colonies^24^ and attempts to manage the nesting of commercial queens in the environment (personal observation). To avoid escapes, bumblebee nests should be placed only inside greenhouses and destroyed at the end of the crop pollination campaign before new sexual individuals emerge. The arrival of commercial breeds of *B. terrestris* into new environments and their subsequent colonization is guaranteed unless stricter regulations on their transport and management are adopted^16^, both outside and within the species’ natural distribution range^8,13^. To our concern, there is still no legislation regarding the management of bumblebee colonies for agricultural use in the Iberian Peninsula beyond the advice and instructions of the provider companies. Because the Iberian Peninsula is a large producer and exporter of fruits and vegetables, and therefore an intensive user of commercial pollination services, we endorse stricter legislation on the importation of foreign subspecies and support for companies breeding local subspecies^11^, as the breeding of *B. t. lusitanicus* does not involve additional costs compared with other subspecies of *B. terrestris* (de Jonghe; Rasmont; personal communication). Such regulations are already in effect in other regions within the natural distribution range of the species, such as the United Kingdom, Norway and the Canary Islands. Authorization of the importation of exotic taxa should only be legalised after studying the survival rate, expansion capacity and environmental impact of the managed populations. It is important to coordinate an update of the existing legislation with more active dissemination to first-hand users on the correct management of commercial breeds and the benefits of using local subspecies.

## Material and methods

### Spatial and temporal framework

Individuals were collected along the Iberian Peninsula, the Pyrenees, north of France, and Belgium (Fig. 1). The sampling was focused on the National Parks of Sierra de Guadarrama and Sierra Nevada (central and southern regions of the Iberian Peninsula, respectively), which are immersed in very different landscape contexts. In this sense, agriculture in the surroundings of Sierra de Guadarrama is more dependent on irrigated and rain-fed crops (greenhouse crops in the surrounding provinces cover less than 200 ha), while in the vicinity of Sierra Nevada, greenhouse crops have expanded over the last 50 years to more than 30,000 ha (Supplementary Fig. S1 Online), some of them less than 20 km from the boundaries of the National Park^38^. To determine the impact of the introduction of commercial bumblebees, a set of individuals collected in Spain in the 1980s before the use of bumblebee colonies started in the peninsula in the 1990s, was included in the analyses. In the current sampling we considered as a population the set of individuals sampled within a radius of 10 km based on the maximum flight distance recorded for *B. terrestris* (according to Kraus et al*.*^57^) taking into account geographic barriers (e.g. if mountains were present).

### Sample collection

A total of 594 individuals were initially selected for this work. From them, 437 individuals were sampled (256 females and 181 males) in a north–south gradient in 17 locations of the Iberian Peninsula (Supplementary Table S1 online). Two sets of reference populations were additionally considered for spatial comparisons: 78 individuals from the Pyrenees as it is the natural intergradation area between the two subspecies, 23 individuals from France (Normandy) and 12 from Belgium as they are within the natural distribution range of *B. t. terrestris*. As a historical reference, 44 individuals collected in Spain before the introduction of commercial *B. terrestris*^39^ were included (Table 1, Supplementary Fig. S7 online).

Individuals from the Iberian Peninsula, Belgium and France were net-sampled and maintained individually in absolute ethanol at -20 ºC until processed. Individuals from the Pyrenees and the historical reference sampling were obtained from the pinned collections of the University of Mons and the Complutense University of Madrid (UCME), respectively. A first subspecies identification of individuals was performed through their morphological characters^18,20^ (i.e. ferruginous brown coloration only present in *B. t. lusitanicus*).

### DNA extraction

Total DNA was extracted from one leg of each individual following Ivanova et al*.*^58^ protocol. Concentration and purity of DNA extractions from pinned individuals were quantified, as DNA obtained from antique individuals presents a lesser quality^29,59^.

### Mitochondrial haplotype screening

Haplotype information of *B. terrestris* individuals was obtained with the RFLP approach on the NADH dehydrogenase subunit 2 gene (*nad2*) following Cejas et al*.*^23^. To improve the amplification success, Bovine Serum Albumin (BSA, 1.2 mg/mL) was added to the PCR reactions.

DNA is often highly fragmented in samples from old collections, making it difficult to amplify larger sequences^59^. Therefore, for the Pyrenean individuals, a new primer set (F: 5’-TCAACATCGAGGTCGCAATCA-3’ and R: 5’-TGGCTGCGGTATAATTGACTGT-3’) was designed with Primer3^60^ to obtain a shorter fragment (388 bp) within the 16S ribosomal RNA (*rrnL*) gene. The same PCR conditions were used as in Hines et al*.*^61^. Amplicons were sequenced in Secugen (Madrid, Spain). We added the haplotype information from the samples processed in Cejas et al*.*^23,39^ by sequencing the *16S* fragment as described in Hines et al*.*^61^.

### Microsatellite amplification and sample genotyping

Ten microsatellite *loci* designed for *B. terrestris*^51,62^ were amplified in all individuals with two multiplex sets following Cejas et al*.*^63^: RB1 (B10, B11, B100, B96, B124 and B126), and RB2 (B118, B119, B121 and B132). PCR products were sent to the Servei Central de Suport a la Investigació Experimental (University of Valencia, Spain) for capillary electrophoresis on an ABI Prism 3700 (Applied Biosystems). Allele scoring was performed using GENEMAPPER 4.8 (Applied Biosystems) by comparing alleles with an internal size standard (GeneScan-500 Liz, Applied Biosystems). A second allele scoring quality control was made manually to avoid the wrong assignation as alleles of fluorescence peaks during the automated allele scoring process. Individuals with missing data > 30% were discarded. Siblings were inferred with Colony 2.0.6.4^64^ with a Full-Likelihood analysis under default parameters for haplodiploid species. We retained only one individual from every kinship with a greater probability of 70%.

### Genetic diversity and effect of hybridization

The individual assignment test was performed with the program GeneClass 2.0^65^ to find the proportion of hybrid individuals among subspecies in the Iberian populations, using Nei’s standard distance^41^ and a Bayesian method^42^ with MCMC method^66^ as the criteria for computation. For these analyses, the purest individuals from reference populations (REF_SP80 for *B. t. lusitanicus*, REF_FR and REF_BL for *B. t. terrestris*) detected in the assignment test (assignation by logarithmic likelihood) in GenAlEx 6.5^67^ were selected as reference groups. The assignment of the Iberian samples was evaluated.

To evaluate the potential effect of the hybridization and introgression between populations, genetic diversity parameters were calculated (1) in Iberian locations with an appropiate number of samples (≥ 14 individuals, 7 sampling locations), (2) in the Iberian Peninsula as one population (SP + PT), and (3) in the four reference populations (dataset recorded as *hIN*). The same genetic diversity parameters were estimated again after removing all detected hybrids (morphologically identified hybridsand thoseindividuals with discrepant mitochondrial haplotype) and naturalized *B. t. terrestris* individuals (dataset recorded as *hOUT*). Same remarks were taken into consideration for the French population, (Supplementary Table S2 online). Finally, to avoid type-I error the analyses were conducted again after removing a random set of the same size (dataset recorded as *rOUT*)*.*

To avoid bias due to the haplodiploidy of the species, only genotypes of female individuals were considered. Micro-Checker 2.2.3^68^ was used to examine genotyping errors such as stutter bands and the frequency of null alleles. Departure of Hardy–Weinberg equilibrium (HWE) and estimates of linkage disequilibrium (LD) were calculated for the Iberian and reference populations using Genepop^69^. The level of significance for the analyses was adjusted with Bonferroni corrections (*p*-value = 0.005 and 0.0002, respectively). Observed and expected heterozygosity (*H*_O_ and *H*_E_) were estimated with GenAlEx 6.5. Allele richness (Ar) and private allelic richness (pAr) were calculated with Hp-Rare 1.1^70^, and rarefacted to a sample size of 10 gene copies. The inbreeding coefficient (*Fis*) was obtained with FSTAT 2.9.4^71^.

Obtained parameters from datasets *hIN* and *hOUT* were compared with the non-parametric Wilcoxon signed-rank test for related samples using package *stats* 3.6.0 (function *wilcox.test*, paired = TRUE) in R^72^. For this test, data was paired according to their label and ranked by their absolute differences. The level of significance for the analysis was adjusted under Bonferroni corrections (*p*-value = 0.0083). Wilcoxon signed-rank test was repeated against the random removal of individuals (datasets *hIN* and *rOUT*), in order to investigate whether the significance of the results was due to the removal of individuals itself or to the identity of those excluded. The Iberian Peninsula as a population (SP + PT) was not included in this analysis to avoid oversampling.The percentage of hybrid individuals found in each location was compared with the values of expected heterozygosity (*H*_E_) and with the change in heterozygosity (Δ*H*_E_), after removing the hybrids (*hOUT─hIN),* to estimate the effect of introgression on the genetic diversity of the populations through linear regressions in R with package *stats* v3.6.2^72^.

### Genetic structure

A clustering analysis was carried out on Structure^73^ with a burn-in period of 100,000 steps and 1,000,000 MCMC repetitions. Potential clustering values (K) 1 to 10 were tested under 10 iterations each. Structure Harvester^42^ was used to select the best fitting K by calculating ΔK^74^. Software Clumpak^75^ was used to reach a consensus between the different iterations of each selected K, following the Markov clustering algorithm. Furthermore, a graphical representation was printed with Distruct 1.1^76^.

Discriminant analyses of principal components (DAPC) was performed using R (R Core Team, 2013) package *adegenet* 2.1.1^77^. Function *find.clusters* was used to identify the number of genetic clusters according to the Bayesian Inference Criterion (BIC). Nevertheless, from a biogeographic perspective, other K numbers were also analysed. Finally, populations were selected as clusters to investigate their identity and relationship. The number of principal components for the analysis was selected after α-score estimations to explain the data while avoiding overfitting. Results were graphically represented in a scatter plot.

### Diploid male monitoring

The proportion (ϕ) of diploid males (2n **♂**) was assessed for each sampling location. Males with two or more diploid *loci* were considered as diploids. Diploidy proportion was calculated following Zayed et al*.*^34^ by dividing the number of diploid males between the total number of sampled male individuals in the location.

## Supplementary Information

Supplementary Information 1.

Supplementary Information 2.

## Data Availability

Microsatellite genotypes of the populations for analysis hIN and hOUT have been detailed in Supplementary Table S3.
