# Supplementary material for: Spatial and temporal patterns of genetic diversity in Bombus terrestris populations of the Iberian Peninsula and their conservation implications
Source: Sci Rep. 2021 Nov 18;11:22471. doi: 10.1038/s41598-021-01778-2 (PMC8602315; doi:10.1038/s41598-021-01778-2)
Supplement: Supplementary file 1 — Supplementary Information 1. [file 41598_2021_1778_MOESM1_ESM.pdf]

# Spatial and temporal patterns of genetic diversity in *Bombus terrestris* populations of the Iberian Peninsula and their conservation implications.

Diego Cejas*[1,2],Pilar De la Rúa[1], Concepción Ornosa[3], Denis Michez[2], Irene Muñoz[1]

[1]Área de Biología Animal, Departamento de Zoología y Antropología Física, Facultad de Veterinaria, Universidad de Murcia, 30100 Murcia, Spain.

[2]University of Mons, Research Institute for Biosciences, laboratoiry of Zoology, Place du parc 20, 7000 Mons, Belgium.

[3]Departamento de Biodiversidad, Ecología y Evolución, Facultad de Ciencias Biológicas, Universidad Complutense, 28040 Madrid, Spain.

*Contact information first author: diegomanuel.cejasacuna@umons.ac.be

# Supplementary Material

**Table S1.** Sampling and geographical information of the Iberian and reference *B. terrestris* populations. Location codes correspond to country and locations (SP=Spain; PT=Portugal). N= number of individuals with the number of females between brackets.

| Code | Location | Country | Latitude (N) | Longitude (W) | N (♀) |
|---|---|---|---|---|---|
| (SP+PT) | | | | | 437 (256) |
| SP_VI | Vitoria | Spain | 42°50'59.96" | 2°40'18.91" | 3 (0) |
| SP_PO | Pontevedra | Spain | 42°19'12.76" | 8°30'19.27" | 11 (7) |
| SP_BU | Burgos | Spain | 42º26´32" | 4º15´50" | 7 (7) |
| SP_PA | Palencia | Spain | 4302´29" | 4º27310" | 5 (5) |
| SP_SO | Soria | Spain | 41° 45' 58" | 2°27'17.20" | 11 (9) |
| PT_BR | Braganza | Portugal | 41°47'56.15" | 6°45'55.72" | 7 (3) |
| PT_VC | Vila do Conde | Portugal | 41°20'38.35" | 8°44'45.85" | 14 (14) |
| SP_SG1 | Sierra Guadarrama | Spain | 40°39'38" | 4°0'41" | 10 (10) |
| SP_SG2 | Sierra Guadarrama | Spain | 40°45'14" | 4°3'50" | 138 (67) |
| SP_SG3 | Sierra Guadarrama | Spain | 40°49'53" | 3°57'34" | 35 (24) |
| SP_MA | Madrid | Spain | 40°26'52.9" | 3°43'34.1" | 12 (9) |
| SP_MU1 | Murcia | Spain | 38° 9'38.72" | 2°13'18.85" | 23 (16) |
| SP_MU2 | Murcia | Spain | 37°52'23.94" | 1°33'42.64" | 25 (18) |
| SP_HU | Huelma | Spain | 37°43'8.75" | 3°27'5.36" | 7 (2) |
| SP_SN1 | Sierra Nevada | Spain | 36°57'54.70" | 3°20'27.13" | 17 (8) |
| SP_SN2 | Sierra Nevada | Spain | 37°10'50.18" | 3° 9'47.24" | 48 (19) |
| SP_SN3 | Sierra Nevada | Spain | 37° 5'36.98" | 3°23'13.13" | 64 (38) |
| REF_BL | | | | | 12(12) |
| | Moorsel | Belgium | 50°57'10.8" | 4°06'21.6"E | 1(1) |
| | Malchamps | Belgium | 50°27'54" | 5°55'22.8"E | 1(1) |
| | Saint-Vaast | Belgium | 50°27'10.80" | 4°7'26.40"E | 9(9) |
| | Torgny | Belgium | 49°31'01.2" | 5°27'54.0"E | 1(1) |
| REF_FR | | | | | 23(18) |
| | Colleville-sur-Mer | France | 49°21'37.86” | 0°51'27.11”W | 1(1) |
| | Beaumont en Auge | France | 49°16'39.58” | 0° 6'33.28”E | 3(3) |
| | Beuvron-en-Auge | France | 49°11'23.1" | 0°02'44.2"W | 6(5) |
| | Chartres | France | 48°29'17.84” | 1°30'47.31”E | 8(4) |
| | Grandcamp-Maisy | France | 49°23'13.53” | 1° 3'8.17”E | 5(5) |
| PN | | | | | 78(78) |
| PN_AM | Argelès-sur-Mer | French Pyrenees | 42°28'27" | 3°00'33"E | 27 (27) |
| PN_EY | Eyne | French Pyrenees | 42°29'12.8" | 02°05'06.4"E | 46 (46) |
| PN_JA | Jaca | Spanish Pyrenees | 42°33'52.50" | 0°34'1.39"W | 5 (5) |
| REF_SP80 | | | | | 44(40) |
| | Salnes | Spain | | | 1(1) |
| | Alsasua | Spain | | | 1(1) |
| | Llanes | Spain | | | 1(1) |
| | Barcelona | Spain | | | 1(1) |
| | Comarruga | Spain | | | 1(1) |
| | Saelices del Río | Spain | | | 1(1) |
| | El Espinar | Spain | | | 1(1) |
| | Ávila | Spain | | | 2(2) |
| | Guadalajara | Spain | | | 1(1) |
| | Budia | Spain | | | 1(1) |
| | Cerceda | Spain | | | 1(1) |
| | Algete | Spain | | | 1(1) |
| | Madrid | Spain | | | 27(24) |
| | Villares del Saz | Spain | | | 1(1) |
| | Mallorca | Spain | | | 1(1) |
| | Cazorla | Spain | | | 1(1) |
| | Marbella | Spain | | | 1(1) |
| TOTAL | (Iberian + Reference) | | | | 594(404) |

**Table S2.** Information on selected individuals for the genetic diversity and structure analyses. For the Iberian populations, only those with 14 or more individuals were selected. Haplotype 1 corresponded to the most frequent in *B. t. lusitanicus* and 2 to the most frequently observed in *B. t. terrestris*. Individuals were marked as potential hybrids in the *analysis_use* column and removed for the *hOUT* analysis when their morphological characters were intermediate between the two subspecies, when their mitochondrial haplotype did not correspond to their subspecies, or when given the geographical distribution of the subspecies, it was suspected that the individual was naturalized.

| Location | ID | subspecies | mit. hapl | analysis_use | sex |
|---|---|---|---|---|---|
| PT_VC | TLPT.010 | lusitanicus | 2 | hybrid | worker |
| PT_VC | TLPT.009 | lusitanicus | 1 | | worker |
| PT_VC | TLPT.008 | lusitanicus | 1 | | worker |
| PT_VC | TLPT.011 | lusitanicus | 1 | | worker |
| PT_VC | TLPT.012 | lusitanicus | 1 | | worker |
| PT_VC | TLPT.013 | lusitanicus | 1 | | worker |
| PT_VC | TLPT.014 | lusitanicus | 1 | | worker |
| PT_VC | TLPT.015 | lusitanicus | 1 | | worker |
| PT_VC | TLPT.016 | lusitanicus | 1 | | worker |
| PT_VC | TLPT.017 | lusitanicus | 1 | | worker |
| PT_VC | TLPT.018 | lusitanicus | 1 | | worker |
| PT_VC | TLPT.019 | lusitanicus | 1 | | worker |
| PT_VC | TLPT.020 | lusitanicus | 1 | | worker |
| PT_VC | TLPT.021 | lusitanicus | 1 | | worker |
| SP_SG2 | TLS.074 | lusitanicus | 2 | hybrid | worker |
| SP_SG2 | TLS.252 | lusitanicus | 2 | hybrid | worker |
| SP_SG2 | TLS.262 | lusitanicus | 2 | hybrid | worker |
| SP_SG2 | TLS.264 | lusitanicus | 2 | hybrid | worker |
| SP_SG2 | TLS.276 | lusitanicus | 2 | hybrid | worker |
| SP_SG2 | TLS.190 | lusitanicus | 1 | | worker |
| SP_SG2 | TLS.253 | lusitanicus | 1 | | worker |
| SP_SG2 | TLS.183 | lusitanicus | 1 | | worker |
| SP_SG2 | TLS.005 | lusitanicus | 1 | | worker |
| SP_SG2 | TLS.070 | lusitanicus | 1 | | queen |
| SP_SG2 | TLS.071 | lusitanicus | 1 | | worker |
| SP_SG2 | TLS.072 | lusitanicus | 1 | | worker |
| SP_SG2 | TLS.073 | lusitanicus | 1 | | worker |
| SP_SG2 | TLS.075 | lusitanicus | 1 | | worker |
| SP_SG2 | TLS.076 | lusitanicus | 1 | | worker |
| SP_SG2 | TLS.153 | lusitanicus | 1 | | worker |
| SP_SG2 | TLS.154 | lusitanicus | 1 | | worker |
| SP_SG2 | TLS.156 | lusitanicus | 1 | | worker |
| SP_SG2 | TLS.157 | lusitanicus | 1 | | worker |
| SP_SG2 | TLS.161 | lusitanicus | 1 | | worker |
| SP_SG2 | TLS.164 | lusitanicus | 1 | | worker |
| SP_SG2 | TLS.165 | lusitanicus | 1 | | worker |
| SP_SG2 | TLS.167 | lusitanicus | 1 | | worker |
| SP_SG2 | TLS.168 | lusitanicus | 1 | | worker |
| SP_SG2 | TLS.169 | lusitanicus | 1 | | worker |
| SP_SG2 | TLS.175 | lusitanicus | 1 | | worker |
| SP_SG2 | TLS.176 | lusitanicus | 1 | | worker |
| SP_SG2 | TLS.178 | lusitanicus | 1 | | worker |
| SP_SG2 | TLS.181 | lusitanicus | 1 | | worker |

| | | | | | |
|---|---|---|---|---|---|
| SP_SG2 | TLS.186 | lusitanicus | 1 | | worker |
| SP_SG2 | TLS.191 | lusitanicus | 1 | | worker |
| SP_SG2 | TLS.203 | lusitanicus | 1 | | queen |
| SP_SG2 | TLS.235 | lusitanicus | 1 | | worker |
| SP_SG2 | TLS.236 | lusitanicus | 1 | | worker |
| SP_SG2 | TLS.237 | lusitanicus | 1 | | worker |
| SP_SG2 | TLS.238 | lusitanicus | 1 | | worker |
| SP_SG2 | TLS.251 | lusitanicus | 1 | | worker |
| SP_SG2 | TLS.255 | lusitanicus | 1 | | worker |
| SP_SG2 | TLS.256 | lusitanicus | 1 | | worker |
| SP_SG2 | TLS.257 | lusitanicus | 1 | | worker |
| SP_SG2 | TLS.259 | lusitanicus | 1 | | worker |
| SP_SG2 | TLS.260 | lusitanicus | 1 | | worker |
| SP_SG2 | TLS.261 | lusitanicus | 1 | | worker |
| SP_SG2 | TLS.265 | lusitanicus | 1 | | worker |
| SP_SG2 | TLS.266 | lusitanicus | 1 | | worker |
| SP_SG2 | TLS.268 | lusitanicus | 1 | | worker |
| SP_SG2 | TLS.269 | lusitanicus | 1 | | worker |
| SP_SG2 | TLS.270 | lusitanicus | 1 | | worker |
| SP_SG2 | TLS.273 | lusitanicus | 1 | | worker |
| SP_SG2 | TLS.275 | lusitanicus | 1 | | worker |
| SP_SG2 | TLS.277 | lusitanicus | 1 | | worker |
| SP_SG2 | TLS.278 | lusitanicus | 1 | | worker |
| SP_SG2 | TLS.279 | lusitanicus | 1 | | worker |
| SP_SG2 | TLS.280 | lusitanicus | 1 | | worker |
| SP_SG3 | THS.008 | hybrido | 1 | hybrid | worker |
| SP_SG3 | TLS.285 | lusitanicus | 2 | hybrid | worker |
| SP_SG3 | TLS.295 | lusitanicus | 2 | hybrid | worker |
| SP_SG3 | TLS.006 | lusitanicus | 1 | | worker |
| SP_SG3 | TLS.007 | lusitanicus | 1 | | worker |
| SP_SG3 | TLS.283 | lusitanicus | 1 | | worker |
| SP_SG3 | TLS.284 | lusitanicus | 1 | | worker |
| SP_SG3 | TLS.286 | lusitanicus | 1 | | worker |
| SP_SG3 | TLS.287 | lusitanicus | 1 | | worker |
| SP_SG3 | TLS.289 | lusitanicus | 1 | | worker |
| SP_SG3 | TLS.290 | lusitanicus | 1 | | worker |
| SP_SG3 | TLS.292 | lusitanicus | 1 | | worker |
| SP_SG3 | TLS.293 | lusitanicus | 1 | | worker |
| SP_SG3 | TLS.294 | lusitanicus | 1 | | worker |
| SP_SG3 | TLS.296 | lusitanicus | 1 | | queen |
| SP_SG3 | TLS.297 | lusitanicus | 1 | | worker |
| SP_SG3 | TLS.301 | lusitanicus | 1 | | queen |
| SP_SG3 | TLS.302 | lusitanicus | 1 | | worker |
| SP_SG3 | TLS.303 | lusitanicus | 1 | | worker |
| SP_SG3 | TLS.305 | lusitanicus | 1 | | worker |
| SP_SG3 | TLS.307 | lusitanicus | 1 | | worker |
| SP_SG3 | TLS.311 | lusitanicus | 1 | | worker |
| SP_MU1 | TLS.326 | lusitanicus | 2 | hybrid | worker |
| SP_MU1 | TLS.328 | lusitanicus | 2 | hybrid | worker |
| SP_MU1 | TLS.338 | lusitanicus | 2 | hybrid | worker |
| SP_MU1 | TLS.344 | lusitanicus | 2 | hybrid | worker |
| SP_MU1 | TLS.343 | lusitanicus | 1 | | worker |
| SP_MU1 | TLS.327 | lusitanicus | 1 | | worker |
| SP_MU1 | TLS.329 | lusitanicus | 1 | | worker |
| SP_MU1 | TLS.331 | lusitanicus | 1 | | worker |
| SP_MU1 | TLS.334 | lusitanicus | 1 | | worker |

| | | | | | |
|---|---|---|---|---|---|
| SP_MU1 | TLS.335 | lusitanicus | 1 | | worker |
| SP_MU1 | TLS.336 | lusitanicus | 1 | | worker |
| SP_MU1 | TLS.339 | lusitanicus | 1 | | worker |
| SP_MU1 | TLS.340 | lusitanicus | 1 | | worker |
| SP_MU1 | TLS.342 | lusitanicus | 1 | | worker |
| SP_MU1 | TLS.347 | lusitanicus | 1 | | worker |
| SP_MU2 | TLS.351 | lusitanicus | 2 | hybrid | worker |
| SP_MU2 | TLS.352 | lusitanicus | 2 | hybrid | worker |
| SP_MU2 | TLS.353 | lusitanicus | 2 | hybrid | worker |
| SP_MU2 | TLS.357 | lusitanicus | 2 | hybrid | worker |
| SP_MU2 | TLS.362 | lusitanicus | 2 | hybrid | worker |
| SP_MU2 | TLS.364 | lusitanicus | 2 | hybrid | worker |
| SP_MU2 | TLS.349 | lusitanicus | 1 | | worker |
| SP_MU2 | TLS.354 | lusitanicus | 1 | | worker |
| SP_MU2 | TLS.355 | lusitanicus | 1 | | worker |
| SP_MU2 | TLS.359 | lusitanicus | 1 | | worker |
| SP_MU2 | TLS.360 | lusitanicus | 1 | | worker |
| SP_MU2 | TLS.365 | lusitanicus | 1 | | worker |
| SP_MU2 | TLS.369 | lusitanicus | 1 | | worker |
| SP_MU2 | TLS.370 | lusitanicus | 1 | | worker |
| SP_MU2 | TLS.371 | lusitanicus | 1 | | worker |
| SP_MU2 | TLS.372 | lusitanicus | 1 | | worker |
| SP_MU2 | TLS.373 | lusitanicus | 1 | | worker |
| SP_SN2 | TTS.014 | terrestris | 1 | hybrid | worker |
| SP_SN2 | TTS.016 | terrestris | 1 | hybrid | worker |
| SP_SN2 | TTS.017 | terrestris | 1 | hybrid | worker |
| SP_SN2 | TLS.106 | lusitanicus | 2 | hybrid | worker |
| SP_SN2 | TLS.108 | lusitanicus | 2 | hybrid | worker |
| SP_SN2 | TLS.399 | lusitanicus | 2 | hybrid | worker |
| SP_SN2 | TTS.015 | terrestris | 2 | hybrid | worker |
| SP_SN2 | TLS.407 | lusitanicus | 1 | | worker |
| SP_SN2 | TLS.104 | lusitanicus | 1 | | worker |
| SP_SN2 | TLS.105 | lusitanicus | 1 | | worker |
| SP_SN2 | TLS.107 | lusitanicus | 1 | | worker |
| SP_SN2 | TLS.109 | lusitanicus | 1 | | worker |
| SP_SN2 | TLS.110 | lusitanicus | 1 | | worker |
| SP_SN2 | TLS.111 | lusitanicus | 1 | | queen |
| SP_SN2 | TLS.112 | lusitanicus | 1 | | worker |
| SP_SN2 | TLS.113 | lusitanicus | 1 | | queen |
| SP_SN2 | TLS.401 | lusitanicus | 1 | | worker |
| SP_SN2 | TLS.403 | lusitanicus | 1 | | worker |
| SP_SN2 | TLS.406 | lusitanicus | 1 | | worker |
| SP_SN3 | TTS.001 | terrestris | 1 | hybrid | worker |
| SP_SN3 | TTS.004 | terrestris | 1 | hybrid | worker |
| SP_SN3 | TTS.006 | terrestris | 1 | hybrid | worker |
| SP_SN3 | TTS.009 | terrestris | 1 | hybrid | worker |
| SP_SN3 | TTS.010 | terrestris | 1 | hybrid | worker |
| SP_SN3 | TLS.080 | lusitanicus | 2 | hybrid | worker |
| SP_SN3 | TLS.433 | lusitanicus | 2 | hybrid | worker |
| SP_SN3 | TLS.434 | lusitanicus | 2 | hybrid | worker |
| SP_SN3 | TLS.438 | lusitanicus | 2 | hybrid | worker |
| SP_SN3 | TLS.440 | lusitanicus | 2 | hybrid | worker |
| SP_SN3 | TTS.002 | terrestris | 2 | hybrid | worker |
| SP_SN3 | TTS.003 | terrestris | 2 | hybrid | worker |
| SP_SN3 | TTS.005 | terrestris | 2 | hybrid | worker |
| SP_SN3 | TTS.007 | terrestris | 2 | hybrid | worker |

| | | | | | |
|---|---|---|---|---|---|
| SP_SN3 | TTS.008 | terrestris | 2 | hybrid | worker |
| SP_SN3 | TTS.013 | terrestris | 2 | hybrid | worker |
| SP_SN3 | TLS.088 | lusitanicus | 1 | | worker |
| SP_SN3 | TLS.439 | lusitanicus | 1 | | worker |
| SP_SN3 | TLS.008 | lusitanicus | 1 | | worker |
| SP_SN3 | TLS.009 | lusitanicus | 1 | | worker |
| SP_SN3 | TLS.010 | lusitanicus | 1 | | worker |
| SP_SN3 | TLS.081 | lusitanicus | 1 | | worker |
| SP_SN3 | TLS.082 | lusitanicus | 1 | | worker |
| SP_SN3 | TLS.083 | lusitanicus | 1 | | worker |
| SP_SN3 | TLS.084 | lusitanicus | 1 | | worker |
| SP_SN3 | TLS.085 | lusitanicus | 1 | | worker |
| SP_SN3 | TLS.087 | lusitanicus | 1 | | worker |
| SP_SN3 | TLS.089 | lusitanicus | 1 | | worker |
| SP_SN3 | TLS.090 | lusitanicus | 1 | | worker |
| SP_SN3 | TLS.091 | lusitanicus | 1 | | worker |
| SP_SN3 | TLS.430 | lusitanicus | 1 | | worker |
| SP_SN3 | TLS.435 | lusitanicus | 1 | | worker |
| SP_SN3 | TLS.441 | lusitanicus | 1 | | worker |
| SP_SN3 | TLS.448 | lusitanicus | 1 | | worker |
| SP_SN3 | TLS.451 | lusitanicus | 1 | | worker |
| SP_SN3 | TLS.452 | lusitanicus | 1 | | worker |
| REF_BL | TTB.001 | terrestris | 2 | | worker |
| REF_BL | TTB.002 | terrestris | 2 | | worker |
| REF_BL | TTB.003 | terrestris | 2 | | worker |
| REF_BL | TTB.004 | terrestris | 2 | | worker |
| REF_BL | TTB.007 | terrestris | 2 | | worker |
| REF_BL | TTB.008 | terrestris | 2 | | worker |
| REF_BL | TTB.009 | terrestris | 2 | | worker |
| REF_BL | TTB.010 | terrestris | 2 | | worker |
| REF_BL | TTB.011 | terrestris | 2 | | worker |
| REF_BL | TTB.012 | terrestris | 2 | | worker |
| REF_BL | TTB.013 | terrestris | 2 | | worker |
| REF_FR | TTF.023 | terrestris | 1 | hybrid | worker |
| REF_FR | TTF.024 | terrestris | 1 | hybrid | worker |
| REF_FR | TTF.026? | terrestris | 1 | hybrid | worker |
| REF_FR | TTF.017 | terrestris | 2 | | worker |
| REF_FR | TTF.001 | terrestris | 2 | | worker |
| REF_FR | TTF.005 | terrestris | 2 | | worker |
| REF_FR | TTF.006 | terrestris | 2 | | worker |
| REF_FR | TTF.009 | terrestris | 2 | | worker |
| REF_FR | TTF.010 | terrestris | 2 | | worker |
| REF_FR | TTF.011 | terrestris | 2 | | worker |
| REF_FR | TTF.012 | terrestris | 2 | | worker |
| REF_FR | TTF.014 | terrestris | 2 | | worker |
| REF_FR | TTF.016 | terrestris | 2 | | worker |
| REF_FR | TTF.018 | terrestris | 2 | | worker |
| REF_FR | TTF.019 | terrestris | 2 | | worker |
| REF_FR | TTF.020 | terrestris | 2 | | worker |
| REF_FR | TTF.022 | terrestris | 2 | | worker |
| PN_EY | TTPN.002 | terrestris | 1 | hybrid | worker |
| PN_EY | TTPN.004 | terrestris | 1 | hybrid | worker |
| PN_EY | TLPN.016 | lusitanicus | 2 | hybrid | worker |
| PN_EY | TLPN.020 | lusitanicus | 2 | hybrid | worker |
| PN_EY | TLPN.025 | lusitanicus | 2 | hybrid | worker |
| PN_EY | TLPN.029 | lusitanicus | 2 | hybrid | worker |

| | | | | | |
|---|---|---|---|---|---|
| PN_EY | TTPN.003 | terrestris | 2 | hybrid | worker |
| PN_EY | TLPN.017 | lusitanicus | 1 | | worker |
| PN_EY | TLPN.018 | lusitanicus | 1 | | worker |
| PN_EY | TLPN.019 | lusitanicus | 1 | | worker |
| PN_EY | TLPN.022 | lusitanicus | 1 | | worker |
| PN_EY | TLPN.024 | lusitanicus | 1 | | worker |
| PN_EY | TLPN.026 | lusitanicus | 1 | | worker |
| PN_EY | TLPN.027 | lusitanicus | 1 | | worker |
| PN_EY | TLPN.028 | lusitanicus | 1 | | worker |
| Ref_80s | TLS.013 | lusitanicus | 2 | hybrid | worker |
| Ref_80s | TLS.011 | lusitanicus | 1 | | worker |
| Ref_80s | TLS.020 | lusitanicus | 1 | | worker |
| Ref_80s | TLS.052 | lusitanicus | 1 | | worker |
| Ref_80s | TLS.012 | lusitanicus | 1 | | worker |
| Ref_80s | TLS.014 | lusitanicus | 1 | | worker |
| Ref_80s | TLS.015 | lusitanicus | 1 | | worker |
| Ref_80s | TLS.016 | lusitanicus | 1 | | worker |
| Ref_80s | TLS.018 | lusitanicus | 1 | | worker |
| Ref_80s | TLS.021 | lusitanicus | 1 | | worker |
| Ref_80s | TLS.022 | lusitanicus | 1 | | worker |
| Ref_80s | TLS.023 | lusitanicus | 1 | | worker |
| Ref_80s | TLS.024 | lusitanicus | 1 | | worker |
| Ref_80s | TLS.027 | lusitanicus | 1 | | worker |
| Ref_80s | TLS.028 | lusitanicus | 1 | | worker |
| Ref_80s | TLS.029 | lusitanicus | 1 | | worker |
| Ref_80s | TLS.030 | lusitanicus | 1 | | worker |
| Ref_80s | TLS.031 | lusitanicus | 1 | | worker |
| Ref_80s | TLS.032 | lusitanicus | 1 | | worker |
| Ref_80s | TLS.035 | lusitanicus | 1 | | worker |
| Ref_80s | TLS.036 | lusitanicus | 1 | | worker |
| Ref_80s | TLS.037 | lusitanicus | 1 | | worker |
| Ref_80s | TLS.040 | lusitanicus | 1 | | worker |
| Ref_80s | TLS.042 | lusitanicus | 1 | | worker |
| Ref_80s | TLS.043 | lusitanicus | 1 | | worker |
| Ref_80s | TLS.046 | lusitanicus | 1 | | worker |
| Ref_80s | TLS.048 | lusitanicus | 1 | | worker |
| Ref_80s | TLS.049 | lusitanicus | 1 | | worker |
| Ref_80s | TLS.053 | lusitanicus | 1 | | worker |
| Ref_80s | TLS.054 | lusitanicus | 1 | | queen |
| Ref_80s | TLS.055 | lusitanicus | 1 | | worker |
| Ref_80s | TLS.057 | lusitanicus | 1 | | worker |
| Ref_80s | TLS.058 | lusitanicus | 1 | | worker |
| Ref_80s | TLS.456 | lusitanicus | 1 | | worker |

**Table S3.** Microsatellite genetic data file in Genepop format of the studied populations. The hIN dataset includes all individuals sampled in the eleven populations, while the hOUT dataset includes the same populations without the individuals described as hybrids according to morphological or genetic criteria.

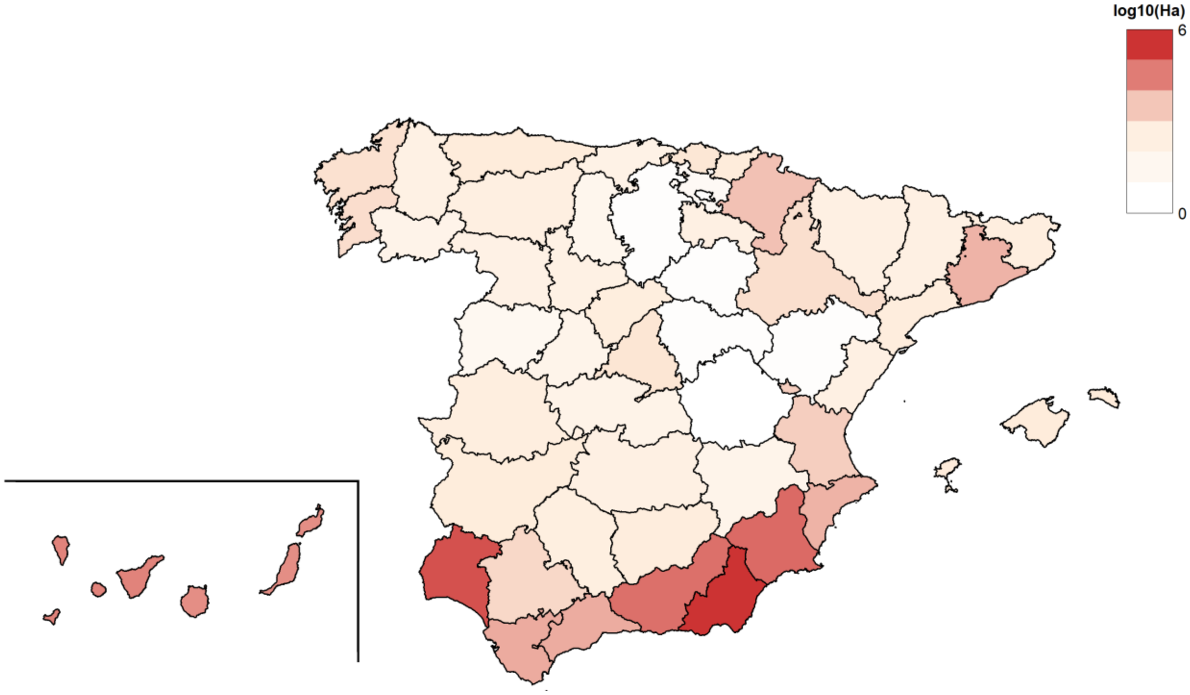


**Figure S1.** Distribution of greenhouses in Spain. Data of greenhouse hectares by province was obtained from the surveys conducted by the Spanish Ministry of Agriculture in 2020[40]. Each province has been coloured with RStudio 1.4 from white to red based on the base ten logarithm of the number of greenhouse hectares in the territory. This map is a derived work from BDLJE 2018 CC-BY 4.0 scne.es.

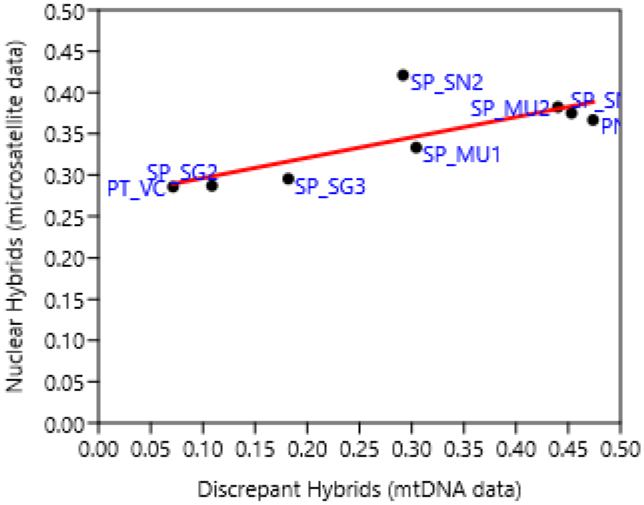


**Figure S2.** Linear regression between the frequencies of discrepant hybrids detected with mtDNA data and the frequencies of nuclear hybrids (microsatellite data) observed in each location ($r^2 = 0.593$, $p= 0.033$). The frequency of nuclear hybrids was obtained as the average of both methods, Nei's standard distance and Bayesian method.

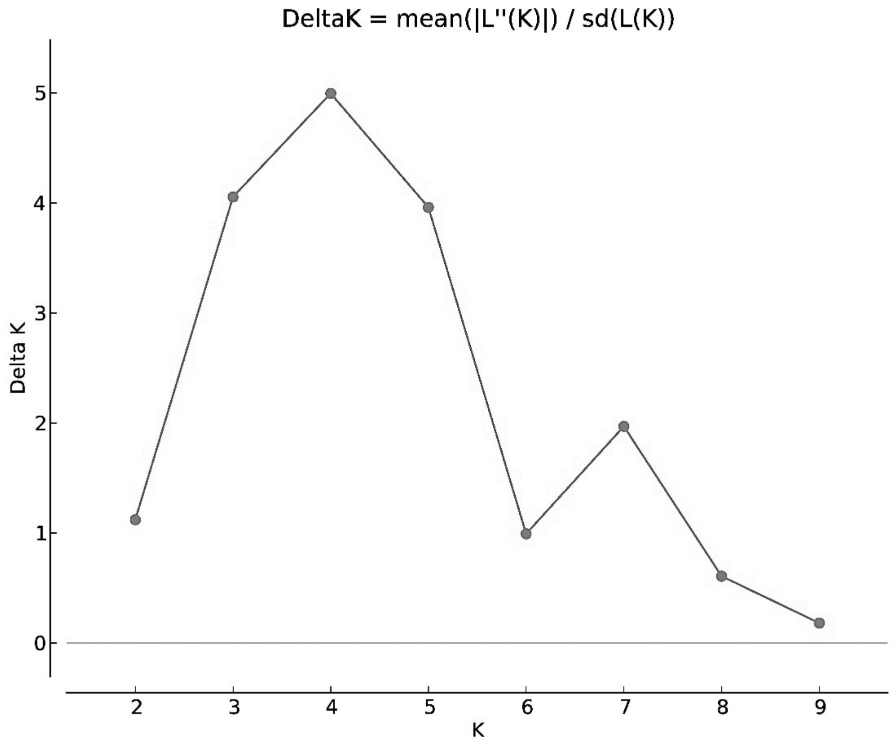


**Figure S3.** ΔK calculated by the Evanno method for models K=1 to K=10.

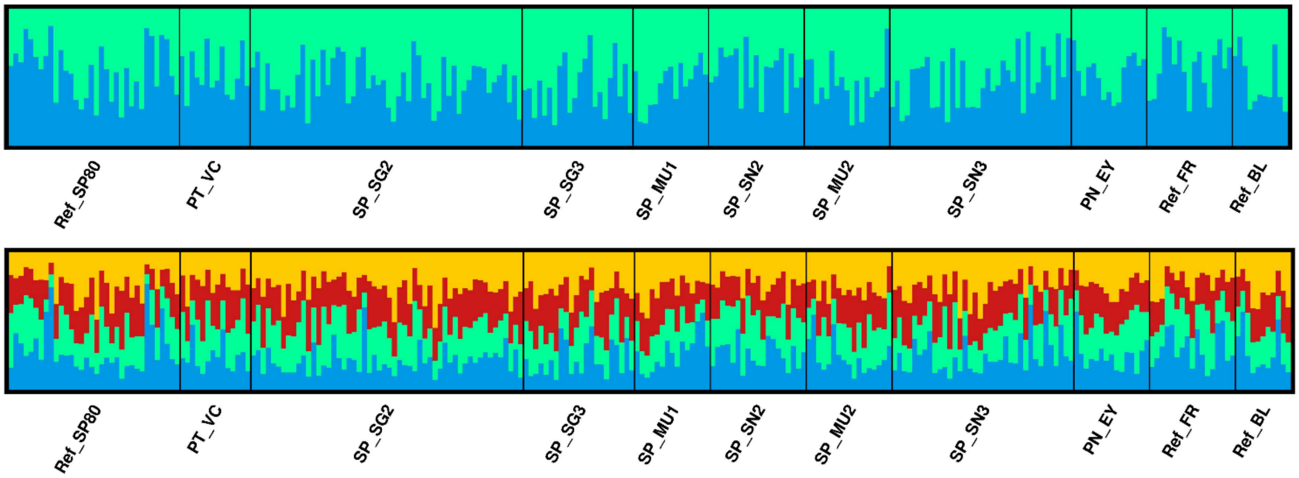


**Figure S4.** Results from STRUCTURE clustering analysis on the microsatellite genotype of 254 females. The consensus of the iterations for K=2 and K=4 after Markov clustering algorithm are shown.

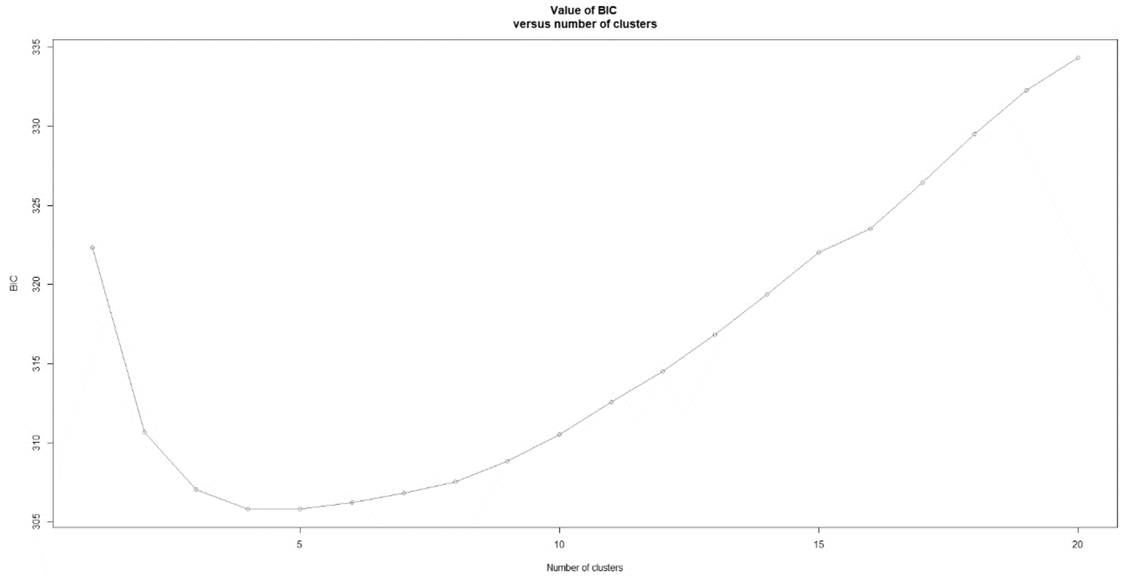


**Figure S5.** Inference of the number of clusters with function *find.clusters* in package adegenet 2.1.1, prior to the discriminant analysis of principal components. Bayesian information criterion (BIC) is provided for every possible number of clusters.

a

b

c

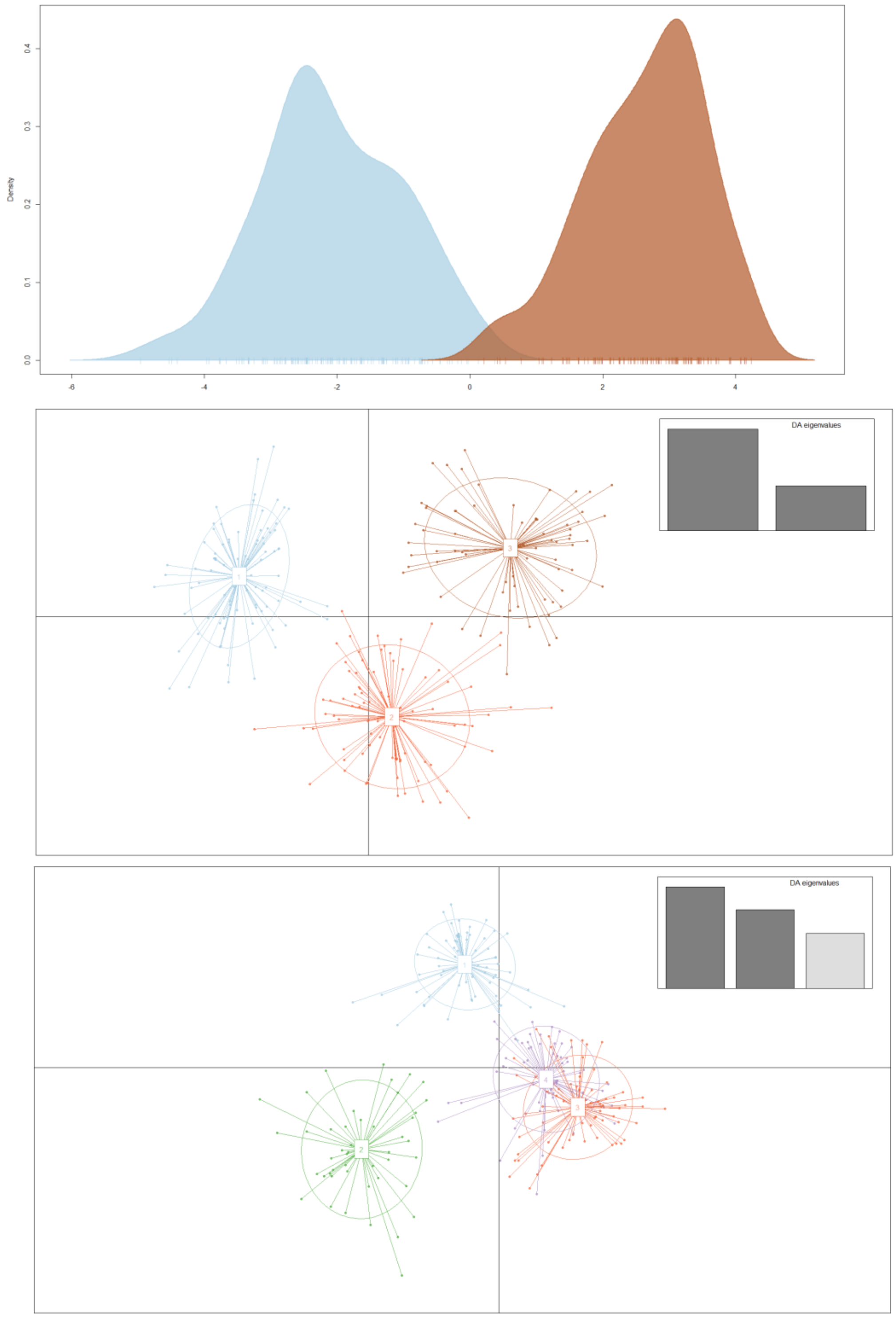


**Figure S6.** Scatter plot from discriminant analysis of principal components based on *find.clusters* results for a) K=2, b) K=3 and c) K=4. Chosen discriminant analysis eigenvalues are depicted in the top right of the plot. Ellipse indicate 95% confidence interval of assignment.

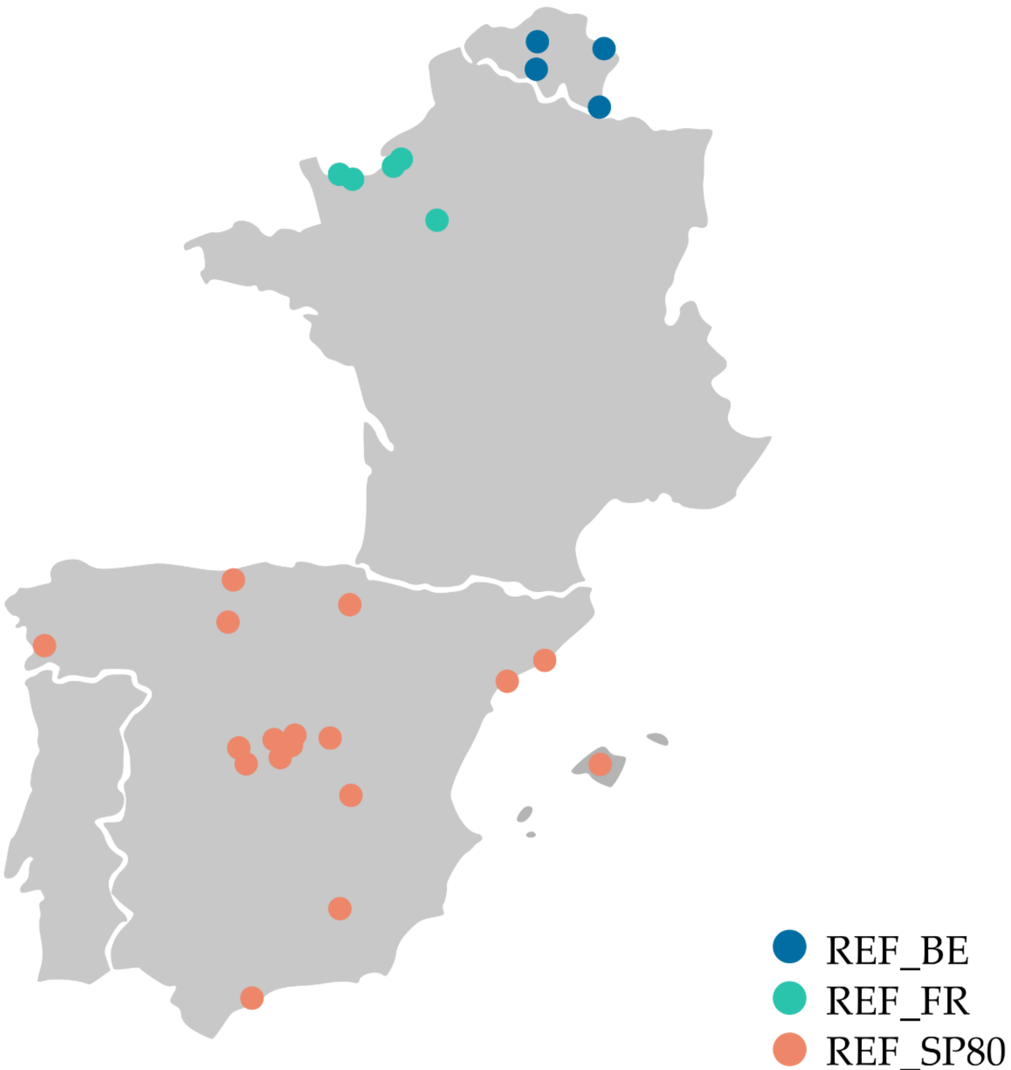


**Figure S7.** Locations of the reference data extracted from Cejas *et al.*[39], as displayed in Table S1. REF_BE in blue colour, REF_FR in green and REF_SP80 in orange. Map adapted from https://freeworldmaps.net/europe/blank_map.html, all rights reserved, with Inkscape 0.92.
